# Nephrotoxicity as a Dose-Limiting Factor in a High-Dose Cisplatin-Based Chemoradiotherapy Regimen for Head and Neck Carcinomas

**DOI:** 10.3390/cancers8020021

**Published:** 2016-02-16

**Authors:** Jantien Hoek, Karen M. Bloemendal, Lilly-Ann A. van der Velden, Judi N.A. van Diessen, Erik van Werkhoven, Willem M.C. Klop, Margot E.T. Tesselaar

**Affiliations:** 1 Department of Head and Neck Surgery and Oncology, Netherlands Cancer Institute, 1066 CX Amsterdam, The Netherlands; j.hoek@nki.nl (J.H.); karenbloemendal@hotmail.com (K.M.B.); m.klop@nki.nl (W.M.C.K.); 2 Department of Otolaryngology and Head & Neck Surgery, Leiden University Medical Center, 2300 RC Leiden, The Netherlands; l.a.van_der_velden@lumc.nl; 3 Department of Radiotherapy, Netherlands Cancer Institute, 1066 CX Amsterdam, The Netherlands; j.v.diessen@nki.nl; 4 Department of Biometrics, Netherlands Cancer Institute, 1066 CX Amsterdam, The Netherlands; e.v.werkhoven@nki.nl; 5 Department of Medical Oncology, Netherlands Cancer Institute, 1066 CX Amsterdam, The Netherlands

**Keywords:** head and neck cancer, nephrotoxicity, chemoradiotherapy, cisplatin

## Abstract

*Purpose*: Loco-regional control and organ preservation are significantly improved with concomitant cisplatin/radiotherapy and are compromised with less than 5% grade 3 nephrotoxicity (creatinine clearance 15–29 mL/min). However, although clinically important, in none of the randomized trials is grade 2 nephrotoxicity (defined as creatinine clearance 59–30 mL/min) mentioned. In this study, we assessed nephrotoxicity in daily practice among patients treated with high-dose cisplatin (100 mg/m^2^ on days 1, 22, and 43), concurrently with chemoradiotherapy (CCRT) and the impact on treatment modifications. *Methods*: 208 patients with advanced-stage malignancies of the head and neck region were evaluated. All patients were treated with high-dose cisplatin CCRT. The main outcome parameters were nephrotoxicity (defined as creatinine clearance grade 2 or more) and cumulative doses of cisplatin and radiation. *Results*: 133 patients (64%) completed all pre-planned courses of cisplatin. Nephrotoxicity was the main reason to discontinue the chemotherapy. Grade 3 nephrotoxicity was seen in 16 patients (8%) while grade 2 nephrotoxicity was seen in 53 patients (25%). Thirty six patients (17%) could not complete the pre-planned chemotherapy due to nephrotoxicity. *Conclusions*: In head and neck cancer patients, nephrotoxicity grade 2 is under-reported but is the major factor for discontinuing cisplatin during CCRT.

## 1. Introduction

Concurrent chemo**-**radiotherapy (CCRT) is the standard of care for unresectable locally advanced head and neck squamous cell cancer (HNSCC), organ preservation, positive surgical margins, and/or lymph node involvement with extra**-**capsular extension [1,2,3]. Platinum-based chemotherapy given concomitantly to radiation (RT) has significant benefits on overall survival and loco**-**regional control [4,5,6,7,8] compared to RT alone. Cisplatin is standardly dosed in a three-weekly schedule of 100 mg/m^2^ (total dose 300 mg/m^2^) combined with standard RT [3,6,7,9,10]. However, a high-dose cisplatin regimen is associated with major toxic side effects (myelosuppression, neurotoxicity, ototoxicity, and nephrotoxicity).

In cisplatin-based CCRT schedules, acute nephrotoxicity is commonly graded by an increase of serum creatinine levels, according to the Common Terminology Criteria for Adverse Events (CTCAE). Using these criteria nephrotoxicity ≥grade 3 occurs in only 4%–9% of patients [7,10,11]. However, Espeli *et al.* [12], for instance used the Risk, Injury, Failure, Loss, End-stage kidney disease (RIFLE) criteria to define renal damage during and after CCRT. In this study 53.7% of all patients developed acute renal failure, which is significantly higher than the 4%–9% grade 3 nephrotoxicity as mentioned in other trials.

In our hospital, we use creatinine clearance as the standard to assess renal function. We consider creatinine clearance a more accurate indicator than serum creatinine levels as it takes bodyweight into account. Nephrotoxicity during CCRT (Glomerular Filtration Rate [GFR] <50 mL/min) may require cisplatin dose reductions. This in turn, however, may lead to poorer treatment outcomes. The effect of cisplatin-induced nephrotoxicity on treatment plan alterations still remains uncertain.

The main purpose of this study is to assess nephrotoxicity in patients treated with CCRT in daily practice and whether this leads to treatment alterations.

## 2. Methods

### 2.1. Patients

The medical records of all consecutive patients with locally advanced HNSCC, who were initially treated with cisplatin CCRT between June 2006 and March 2011 were analyzed. All patients had histologically and/or cytologically proven primary HNSCC (T1-4), M0 status, and a CCRT indication with curative intent. Exclusion criteria were chemotherapy with different or combined agents, or low-dose (6 mg/m^2^) cisplatin treatment schedules.

### 2.2. Ethical Considerations

This study does not fall under the scope of the Medical Research Involving Human Subjects Act (WMO), which means that it does not have to be reviewed by an accredited MREC.

### 2.3. Chemotherapy

Cisplatin CCRT regimen comprised of hospital admission with intravenous cisplatin (100 mg/m^2^ on days 1, 22, and 43 during radiotherapy) and is the standard care in our hospital. Patients are pre-hydrated 12 h prior to treatment, and posthydrated for 2 days with at least 3.5 L of 0.9% saline with 60 mmol/L of KCl and 30 mmol/L of MgSO_4_, as well as anti-emetics (aprepitant). Contraindications for high dose cisplatin were patients in a poor state of general and mental health, initial GFR <60 mL/min, and patient preference for an outpatient treatment setting, in which case a low-dose cisplatin (daily 6 mg/m^2^) was generally offered.

### 2.4. Radiotherapy

Cisplatin was concurrently given with conventional fractioned RT (10 gy in 5 daily fractions). A radiotherapy plannings CT-scan was done on which the primary tumor, pathologic lymph nodes and elective nodal areas were delineated. Patients were treated with 6- or 10-MV photons . The elective nodal regions (bilateral level II-V and level I in case of oral cavity tumors) and the primary tumor were irradiated to 46 Gy in 23 fractions. This was immediately followed by a boost of 24 Gy in 12 fractions to a total dose of 70 Gy to all involved regions. In the postoperative setting where macroscopic tumor was suspected in case of irradical resection, the maximum boost dose was 66 Gy (20 Gy in 10 fractions) to regions. In case of microscopic spread a boost was given with seven fractions of 2 Gy to a total dose of 60 Gy. A boost dose of 20 Gy (10 Gy in five fractions) to a total dose of 54 Gy was given to tumors which were radically excised but with close tumor margins.

### 2.5. Evaluation

Patient evaluation took place twice weekly, or more frequently if necessary, and included laboratory tests for Hemoglobin, Hemaocrit, leukocytes, platelets, creatinine, and electrolytes. Patients were intensively instruated to maintain sufficient intake. Specialized dietitians monitored intake and weight loss, and speech therapists were available to appraise the swallowing function during and after treatment.

### 2.6. Renal Function

Renal function is expressed in both creatinine clearance and serum-creatinine levels. The Cockcroft-Gault formula [13] was used to calculate glomerular filtration rate (GFR). Nephrotoxicity was graded according to the CTCAE version 4.0 (chronic kidney disease) defined as grade 2 (GFR between 59–30 mL/min), grade 3 (GFR between 29–15 mL/min) or grade 4 (a GFR <14 mL/min; dialysis or renal transplant indicated). In our hospital, whenever GFR falls below 50 mL/min, chemotherapy is discontinued or adjusted in dosage. Grade 2 nephrotoxicity was therefore sub-divided into one group with clinical consequences (GFR 49–30 mL/min) and one group without clinicial consequences (GFR 59–50 mL/min). Renal function was also expressed as serum-creatine levels according to CTCAE version 4.0 for acute nephrotoxicity. Grade 1 is defined as a creatinine level increase >0.3 mg/dL or creatinine 1.5–2.0× above baseline, grade 2 is defined as creatinine 2–3× above baseline, grade 3 as creatinine >3× above baseline or more than 4.0 mg/dL and grade 4 as having life threatening consequences; dialysis indicated. Evaluation of renal function took place the day before every cisplatin infusion, 4 consecutive weeks after the last infusion, 3 months after the final infusion and during the last documented follow-up visit.

### 2.7. Food and Fluid Intake

It is policy in our institute to encourage oral intake during treatment, to keep the swallowing mechanism active in order to reduce fibrosis and increase the post-treatment swallowing ability. Therefore, tube placement is not a standard procedure prior to therapy. Nasal gastric tube (NGT) or percutaneous radiological gastrostomy (PRG) were placed if weight loss >10% occurred or if food and/or fluid intake was insufficient.

### 2.8. Statistical Analyses

The primary objective of the study was to evaluate nephrotoxicity during and after treatment with cisplatin-based CCRT and to evaluate the number of treatment alterations of cisplatin due to nephrotoxicy. In addition a comparison was made between the incidence of nephrotoxicity for creatinine clearance and serum creatinine levels. Since the GFR was not normally distributed, we reported median and interquartile ranges (IQR). All analyses were done in R version 2.15.3 (http://www.r-project.org/).

## 3. Results

### 3.1. Patient Characteristics

Between June 2006 and March 2011, 320 patients were treated with CCRT for locally advanced HNSCC. Patients were excluded from this study if treatment consisted of low-dose infusions (77 patients), neoadjuvant poly-chemotherapy (18 patients), or combined agents (6 patients). Data was incomplete for 11 patients. Two-hundred-and-eight patients remained for the quantitative analysis. The specific patient characteristics are listed in Table 1. Median follow up was 26 months (range 1–68).

**Table 1:** Patient characteristics (High Dose CCRT N = 208).

Sex	
Male	152 (73%)
Female	56 (27%)
Age	
Median (range)	59 (32–79)
Mean (sd)	58 (8.9)
Tumor location	
Oral cavity	24 (12%)
Oropharynx	106 (51%)
Nasopharynx	17 (8%)
Hypopharynx	40 (19%)
Larynx	10 (5%)
Paranasal sinus	9 (4%)
Parotic gland	2 (1%)
Tumor-stage *	
T1	25 (12%)
T2	55 (26%)
T3	65 (31%)
T4	62 (30%)
Tx	1 (0%)

* The tumor stages were defined according to the American Joint Committee on Cancer Staging for Head and Neck Cancer criteria 1997 [13]. Tx = no tumor staging possible. Abbreviations: CCRT = concurrent chemoradiotherapy, sd = standard deviation.

### 3.2. Treatment Characteristics

Table 2 lists the main treatment characteristics, including treatment intent, cisplatin courses administered and intake method during treatment.

**Table 2:** Treatment characteristics (High Dose CCRT N = 208).

Treatment Intent	N (%)
Definitive	189 (91%)
Postoperative	19 (9%)
Cisplatin courses	
1	15 (7%)
2	63 (30%)
3	130 (63%)
Mean cisplatin dose mg/m^2^ (sd)	254 (62.3)
Radiotherapy Median total dose Gy (sd)	70

Abbreviations: CCRT = concurrent chemoradiotherapy, sd = standard deviation.

**Figure 1:**
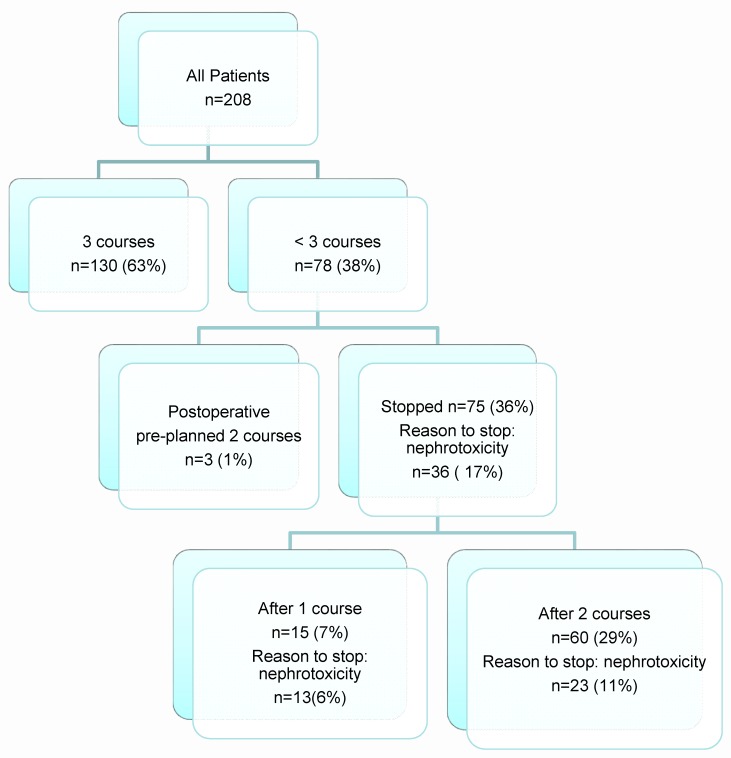
Treatment characteristics summarized.

The flow-chart of Figure 1 outlines how many patients completed the several courses of cisplatin. One hundred and thirty patients (63%) completed all three preplanned courses of cisplatin, although nine of them needed dose reduction in their second or third courses due to renal or hematological toxicity. These nine patients received 89% of their total pre**-**planned cisplatin doses. Seventy-eight patients of this cohort did not complete three courses of cisplatin. For three postoperative patients, only two courses had been planned. These patients were retrieved for study analysis of dose reduction. In all, 75/208 patients (36%) could not complete their pre-planned cisplatin schedules; 15 of these patients received only one course and 60 received two courses.

Nephrotoxicity was the main reason for a dose reduction in 36/208 patients (17%); 13 (6%) had to stop after one course, 23 (11%) after two courses. We did not find any significant predictors (*i.e.*, comorbidity, smoking status) for the development of nephrotoxicity. Other reasons to stop were hematologic toxicity (16/208), severe infection/sepsis (5/208), patient request (6/208), ototoxicity (3/208), nausea/vomiting (2/208), cardiovascular problems (2/208), PRG-related complications (2/208), and death during treatment (3/208).

Ninety-six percent of all patients received full radiotherapy doses with a median of 70 Gy. Reasons for not completing radiotherapy were death during treatment (3/208), severe infection (3/208) or patient request (2/208).

### 3.3. Evaluation of Renal Function

Renal function was evaluated from the start of CCRT and up to 3 months after treatment. In this period, five patients died: two during treatment (from cardiovascular disease); three after treatment but before tumor response evaluation at 3 months (two from progression of distant metastasis and one from cardiovascular disease). One patient was lost in follow-up before the 3-month evaluation date. Table 3 outlines the nephrotoxicity during treatment. After one course, grade 2 nephrotoxicity expressed in GFR 59–50 mL/min had developed in 10/208 (5%) patients and after two courses in 5/193 (2%). Grade 2 nephrotoxicity expressed in GFR 49–30 mL/min developed in 12/208 (6%) patients after one course, and 26/193 (13%) after two courses. Three months after their last infusions, 44/208 (21%) of patients still had nephroxicity grade 2 or higher; and four patients (2%) had grade 3 (GFR 29–15 mL/min).

**Table 3:** Grades of nephrotoxicty.

	GFR	Serum	GFR	Serum Creatinine
	59–30 mL/min →59–50 mL/min * →49–30 mL/min **	creatinine 2–3× above baseline	<29–15 mL/min	>3× above baseline or creatinine increase >35 µmol/L
	Grade 2 ***	Grade 2 ***	Grade 3 ***	Grade 3 ***
**Before therapy N = 208**	0 (0%)	0 (0%)	0 (0%)	0 (0%)
**After 1 course N = 208**	22 (11%) →10 (5%) →12 (6%)	3 (1%)	8 (4%)	4 (2%)
After 2 courses N = 193 ****	31 (16%) →5 (2%) →26 (13%)	8 (4%)	8 (4%)	7(4%)
After 3 months N = 202 *****	42 (21%) →12 (6%) →30 (14%)	14 (7%)	4 (2%)	4 (2%)

* If GFR was <50 mL the next course was cancelled; ** Clinical**ly** relevant because < 50mL the next course was cancelled; *** Following CTCAE version 4.0; **** 15 patients only received 1 cisplatin course; ***** 6 patients died before 3 months of follow-up; Abbreviation: GFR = glomerular filtration rate.

If nephrotoxicity was expressed as serum creatine levels only 3/208 (1%) had developed grade 2 nephrotoxicity after the first course; 8/193 (4%) had developed grade 2 nephrotoxicity. After 3 months 14/202 (7%) had developed a grade 2 and 4/202 (2%) grade 3 nephrotoxicity.

Table 4 outlines renal function in patients before and after treatment. It demonstrates a significant difference (*p* = 0.0008) between the start GFR of patients that stopped the cisplatin after one or two courses due to nephrotoxicity, compared to the patients that did not stop.

**Table 4:** Glomular filtration rate in mL/min during and after treatment.

	All Patients N = 208	Stop Chemotherapy after 1 Course, Due to Nephrotoxicity N = 13	Stop Chemotherapy after 2 Courses, Due to Nephrotoxicity N = 23
Start: GFR median (range)	101( 57–235)	88 (60–119)	84 (62–124)
After 3 months: GFR	80 (4–225)	68 (27–111)	88 (28–203)

Abbreviations: GFR = glomular filtration rate.

## 4. Discussion

In loco-regionally advanced HNSCC, the standard of treatment is CCRT with three courses of single agent cisplatin 100 mg/m^2^. Apart from its high effectiveness as an antitumor agent and a radio-sensitizer, cisplatin is known for its toxic side effects, such as nephrotoxicity, neurotoxicity, and ototoxicity [14]. Due to these toxic side effects, 36% of our study cohort patients could not complete their three pre-planned courses of cisplatin. In 17% of all patients, nephrotoxicity was the main reason to discontinue cisplatin chemotherapy.

Acute nephrotoxicity has been described in previous publications varying from 1% to 46% [7,9,10,12]. Forastiere *et al.* [7] reported grade 3–4 nephrotoxicity in only 4% (CTCAE version 1.0), and Rades *et al.* [10] in 8% (CTCAE version 2.0). These numbers are comparable to the grade 3 nephrotoxicity (8%) in our study. In contrast to our method, the above authors used “increase in serum creatinine” as a measure for renal dysfunction, instead of GFR. In our opinion, GFR calculated with the Cockcroft and Gault formula, is a more accurate indicator for renal function, because bodyweight is taken into acount. This makes a substantial difference, especially in the HNSCC population: many of these patients have below-average body weight and muscle mass due to addiction to nicotine and alcohol. In such populations, nephrotoxicity can be under**-**graded if expressed in terms of creatinine values alone. This might explain the higher incidence of nephrotoxicity we found.

In our study using GFR as a measure for renal function, acute nephrotoxicity grade 2 was found in 11% of patients after the first course and in 16% after the second course of cisplatin. By comparison, when expressing nephrotoxicity in the same study population using serum creatinine levels, we observed a lower incidence of nephrotoxicity grade 2 in 1% and 4% after the first and second course**s** of cisplatin respectively. This streng**t**hens the argument for the use of GFR as a measure for renal function instead of serum creatinine levels, as mentioned above.

In the literature however, grade 2 nephrotoxicity is scarcely reported. In our clinic a GFR below 50 mL/min (grade 2) is considered to be a contraindication for administering the next cisplatin infusion. We therefore state that the prevalence of acute nephrotoxicity is underestimated and the effect on treatment is much greater than reported. The clinical importance of grade 2 is furthermore supported [12,15] with published figures of up to 46% of patients not receiving all three pre**-**planned doses of cisplatin due to treatment related toxicity. We assume that a significant number of these patients will have a dose reduction due to grade 2 nephrotoxicity. The incidence of grade 3–4 nephrotoxicity in our study was similar to that reported in other randomized studies for both creatinine clearance and serum-creatine.

In our study, 17% of the patients could not complete their pre-planned cisplatin treatment schedules due to nephrotoxicity. The impact of treatment plan alterations after one or two courses on loco-regional tumor response and OS remains unclear. However a recent systematic review assessing optimal cisplatin dosage suggests a cumulative dose of at least 200 mg/m^2^ cisplatin [16]. As we speculate that dose reduction leads to decreased loco-regional survival, more kidney damage therefore seems acceptable but it is more important to minimize the toxic side effects of cisplatin. Different options to minimize toxic side effects have been discussed in literature.

Firstly, reduction of toxicity might be achieved by adjusting cisplatin schedules [9,10,12,17]. Espeli *et al.* [12] compared a 3-weekly 100 mg/m^2^ schedule with a weekly 40 mg/m^2^ cisplatin schedule, reporting less renal failure (*p* = 0.04) for patients receiving a weekly schedule. Rades *et al.* [10] also reported diminished renal toxicity in cisplatin schedules with daily 20 mg/m^2^ on day 1–5 and day 29–33 (1% grade 3 nephrotoxicity), compared to 3-weekly 100 mg/m^2^ scheduling (8% grade 3 nephrotoxicity). Furthermore, the important role of (hyper)hydration and diuresis in nephron-protection is considered to be well known [14,18]. During treatment patients lose weight and/or become volume depleted during radiation predisposing to pre-renal azotemia, which might contribute to acute kidney injury alone or in combination with chemotherapy. To ensure proper hydration and nutritional status during chemoradiation, patients may require tube feeding [19] during their treatment period. PRG placement before initiating treatment might therefore indirectly contribute to reducing nephrotoxicity. In our institution placement of a PRG is not standard approach at start of treatment, and it is only placed when there is an indication during treatment. There is a lack of evidence about which approach is preferred comparing advantages and adverse effects of PRG placement. Finally, new medication for nephroprotection is also a continuous subject of investigation in the pharmaceutical industry. Also, it is important to minimize medication for comorbidities prior to treatment, as this might predispose to kidney injury. Nevertheless, the use of agents other than prior saline infusion alone (mannitol, furosemide) remain controversial and do not overcome the nephrotoxicity of cisplatin [18,20,21,22]. *In vitro* and animal testings of new nephroprotective agents have shown encouraging results, but these remain preliminary findings [14,23,24].

We conclude that in HNSCC patients, nephrotoxicity measured as GFR is the major limiting factor for cisplatin continuation during CCRT, leading to treatment alterations in a significant number of patients. Further research is necessary to evaluate whether cisplatin omission during chemo-radiation leads to reduced survival and therefore acceptance of increased nephrotoxicity.
